# Homes for Inebriates

**Published:** 1907-12-28

**Authors:** 


### HOMES FOR INEBRIATES.

### PLAS-YN-DINAS, DINAS MAWDDWY, MERIONETHSHIRE.

Leaving Euston at 11 a.m., and taking care to get into a Cambrian carriage, the traveller will find himself at Cemmes Road about 4.30 p.m.; and from this little wayside station he will have an eight-mile drive up one of the loveliest valleys in the British Isles. The road passes through Cemmes Road village, crosses the boundary between Montgomeryshire and Merionethshire; a few miles further on is Mallwyd, and at the end of the next village, Dinas Mawddwy, is the entrance to Plas-yn-Dinas. From the gates to the house is quite a short distance. The mansion is about forty years old, and is a fine specimen of modern Elizabethan. The rooms are large and are handsomely furnished; and there is an air of spaciousness, and almost of grandeur, about the place, which is rare in an institution of any kind. In fact, it was built literally regardless of expense as a private residence. It is now licensed for fourteen patients, is intended solely for the upper classes, and men only are admitted.

Dr. Walker receives voluntary cases, but does not encourage this class of patients, as experience has shown him that they are not so likely to turn out so well as those placed under the Act, and this is precisely what might be expected by anyone conversant with the disease. It is therefore very desirable, perhaps almost imperative, that those who really wish to be cured should place themselves under the provisions of the Act for at least one year, and we should advise even a longer period, although patients may be received at Plas-yn-Dinas for six months.

The important question is, In what proportion of cases can cures of the drink or drug habit be looked for? Dr. Walker thinks, and few men have had greater experience in the treatment of alcoholism than he, that about twenty-five per cent. of his patients are cured. When it is known that a large number of his cases are almost hopeless when placed under his care—hopeless by reason of degeneration of some part of the nervous system—and that he does not include anyone among the recoveries until two years have passed subsequent to the date of leaving the institution, then this percentage must be deemed a very creditable one indeed. Early treatment is the great essential.

As regards purely medical treatment, Dr. Walker says that many medicines are useful in promoting recovery, but that he has not yet found a specific. Referring to the nostrums so widely advertised nowadays, Dr. Walker remarked to us that analysis had satisfied him that while some of these preparations were innocent and useless, others were harmful and dangerous. It is, however, to be feared that no warning will prevent some deluded people from wasting their money, and what is worse, their time, on these quack medicines.

The public rooms are most cheerful, the library contains four thousand volumes, and there is a billiard-room. Immediately surrounding the mansion are about forty acres of pleasure-grounds and lawns. There is a home farm of two hundred acres; and this, again, is surrounded by an estate of something like 30,000 acres, over which the shooting is carefully preserved for the use of the patients; and there are also about twenty-four miles of fishing. Except hunting, every variety of sport may be indulged in; and the artist could find many pretty bits for his brush. The second highest mountain in Wales is on the estate, and it is flanked by many smaller hills. The inhabitants of this part of Wales have likened it to Switzerland, and verily there is some justification for the comparison.

We have already said that Plas-yn-Dinas is for the upper classes only; and the charge is six guineas a week, which sum includes all medical treatment except high-pressure electricity, which is used in some cases of neurasthenia, and in the earliest stage of locomotor ataxia.

Patients' friends are allowed to visit them, and they can stay at the Peniarth Arms in Mallwyd, a mile or two from Plas-yn-Dinas, at which a covered carriage may be ordered to meet the traveller at Cemmes Road, unless he be satisfied with an open conveyance, which may be obtained by applying at the Dovey Hotel, exactly opposite the Cemmes Road station.

